# Reducing Occurrence of *Giardia duodenalis* in Children Living in Semiarid Regions: Impact of a Large Scale Rainwater Harvesting Initiative

**DOI:** 10.1371/journal.pntd.0002943

**Published:** 2014-06-19

**Authors:** Jacqueline Evangelista Fonseca, Mariângela Carneiro, João Luiz Pena, Enrico A. Colosimo, Nívea Bispo da Silva, André Gabriel F. C. da. Costa, Luciano E. Moreira, Sandy Cairncross, Léo Heller

**Affiliations:** 1 School of Engineering; Department of Sanitary and Environmental Engineering – Universidade Federal de Minas Gerais, Belo Horizonte, Minas Gerais, Brazil; 2 Department of Parasitology; Institute of Biological Sciences and Graduate Program in Health Sciences: Infectious Diseases and Tropical Medicine – Universidade Federal de Minas Gerais, Belo Horizonte, Minas Gerais, Brazil; 3 Department of Statistics; Institute of Mathematical Sciences – Universidade Federal de Minas Gerais, Belo Horizonte, Minas Gerais, Brazil; 4 Laboratory of Parasitology – Universidade Presidente Antônio Carlos, Teófilo Otoni, Minas Gerais, Brazil; 5 London School of Hygiene and Tropical Medicine, London, United Kingdom; Emory University, United States of America

## Abstract

**Background:**

In Brazil, about two million people living in rural semiarid regions were benefited with the construction of rainwater cement cisterns, as an initiative from the program “One Million Cisterns” (P1MC). Nevertheless, few epidemiological studies have been conducted to assess health risks or protection effects associated with consumption of this water source. The aim of this study was to evaluate whether access to rainwater harvesting cisterns is associated with the decrease in the occurrence of *Giardia duodenalis* infections in children, compared to other children living in households supplied by other water sources.

**Methodology/Principal Findings:**

A quasi-experimental study with two concurrent cohorts was developed in two rural municipalities of the semiarid region of Brazil. A sample of 664 children, aged between 4 months and 5 years old, was followed up, of which 332 had access to rainwater cisterns (cistern group) and 332 did not, having water supplied from alternative sources (comparison group). In a period of approximately one year (2010) intestinal parasites were investigated in feces three times. The prevalence of *G. duodenalis* in children from the cistern group ranged from 4.8 to 10.5%, while the prevalence in the comparison group ranged from 7.6 to 16.7%. Multivariate analysis (GEE) showed a higher risk of *G. duodenalis* infection in children who did not have access to rainwater cisterns, when compared to children who did (OR 1.72; 95% CI 1.14–2.59). The other variables associated with *G. duodenalis* infection were: number of rooms per house (OR 0.89; 95% CI 0.80–0.99); family income (OR0.48; 95% CI 0.26–0.88); birth order (OR 1.72; 95% CI 1.17–2.51); preterm children (OR 1.70; 95% CI 1.19–2.43); and improper hand hygiene prior to food preparation (OR 4.78; 95% CI 1.95–11.76).

**Conclusions/Significance:**

Ownership of a rainwater cistern is associated with a lower prevalence of *G. duodenalis* infection in children after adjustment for environmental and family-related factors. Nevertheless, the study suggests the necessity to complement physical interventions with actions related to personal and domestic hygiene to enable further reductions in parasite infections affecting mainly the underprivileged populations.

## Author Summary

Currently, rainwater harvesting cisterns built up through the “One Million Cisterns” Program, coordinated by the Brazilian Government, represents the most important public policy for water supply in the rural semiarid region of Brazil. Despite the extensive use of this water provision, few studies have been performed to assess the associated health impact. To assess such impact, this study investigated factors associated with the occurrence of *Giardia duodenalis* (protozoan closely related to the ingestion of contaminated water) in children aged between 4 months and five years old. Two distinct groups of children, one with access to rainwater cisterns (cistern group) and the other without cisterns, with water supplied from alternative sources, including rivers, springs or dams (comparison group) were followed up for approximately one year. It was observed that the risk occurrence of *G. duodenalis* prevalence was greater in children from the comparison group, when compared with those from the cistern group, indicating that the odds of infection by this parasite were 1.72 times greater for comparison group children. In conclusion, the study showed health benefits related to rainwater cisterns, but also identified that other complementary sanitation interventions are needed to minimize the risk of disease in populations with limited access to drinking water.

## Introduction

Intestinal infections caused by *Giardia duodenalis* (syn. *G. intestinalis*, *G. lamblia*) involve several different mechanisms of transmission and risk factors [1]–[3]. These include poor conditions of water supply and sanitation, which explain the higher prevalence of this parasite in developing countries in which access to water supply, sanitation and health services is less widespread and less efficient. Giardiasis is still considered a neglected disease by the World Health Organization, despite its significance and scope, since it occurs primarily in the poorest regions, has a negative impact on child health, pregnancy and worker productivity, and impairs the ability of those infected to achieve their full potential, affecting their socio-economic development [4], [5].

In Brazil, such intestinal parasitic infections are still a significant public health problem, despite the reversal of the mortality profile in recent decades. Infectious and parasitic diseases were the eighth most significant category of causes of death in Brazil in the year 2008, representing 4.4% of the total [6]. Enteric infections can have a long-term impact on child growth and development, and Giardia in particular has been shown to have an impact even when it is not causing any diarrhea at all (5).

Based on research in Brazilian communities living in poor conditions with inadequate or insufficient health and sanitation services, the prevalence of intestinal parasites ranged from 53% to 83%, while the prevalence of *G. duodenalis* ranged from 11% to 32% [7]–[10]. In all of these studies, the authors related the high prevalences found to the poor hygiene and water and sanitation conditions, including access to water with improper quality.

One of the forms of *G. duodenalis* transmission is the intake of water or food contaminated with protozoan cysts, which are released in the host's feces. Heller et al. [11] reported high concentrations of Giardia cysts in sanitary sewers and in water supply sources in Brazil that were contaminated by human feces and feces from animals surrounding the river basin. Such factors favor the risk of *G. duodenalis* infection, associated with water consumption. In rural areas, the situation is even more critical, considering the difficulties in accessing treated water in sufficient amounts to maintain proper hygienic conditions. In Brazil, 72% of the population living in rural areas, or approximately 21 million inhabitants, relies on alternative supply sources, such as rivers, springs and dams because they are not connected to the main water supply network [12].

In Brazilian semiarid regions, where the dry season lasts approximately eight months, the “Programme for One Million Cisterns” (P1MC) was created to enable a better quality of life for rural families, which are the ones most affected by drought. The programme was developed in 1999 by civil society organizations, which had formed a coalition known as the ASA (Articulação do Semiárido Brasileiro). Its primary goal is to build one million cement cisterns for the storage of rainwater collected through gutters attached to the roofs of homes.

In municipalities where P1MC is implemented, municipal commissions are formed by public managers, representatives of rural communities and ASA. These commissions select the families to receive the cisterns based on pre-established criteria. Families from the rural zone of the Brazilian semiarid region with no drinkable water in the surroundings of their household or with unreliable existing sources are considered top priority. The remaining criteria are evaluated in the following order [13]: (1) per capita monthly family income up to half a minimum wage (approximately USD 339), or whose total income is up to three times the minimum wage a month (approximately USD 1,017); (2) female-headed households; (3) greatest number of children less than 6 years old; (4) greatest number of children of school age; (5) greatest number of disabled people; (6) greatest number of elderly.

Each selected family receives a cistern with a capacity to store 16,000 liters of water, which, according to the ASA, is sufficient for a family composed of 5 people to drink, cook and brush their teeth during the dry season. By mid-2012, more than 418,000 cisterns had been built, which benefited approximately 2.1 million inhabitants of semiarid regions [14].

Although rainwater is recognized as a purer source of water [15], various geographic [16], [17], atmospheric [18], harvesting surface-related [19]–[23] and cistern management-related [15], [20], [24], [25] factors may impair its quality and, consequently, pose a risk to the health of populations that use the rainwater, especially for drinking.

Studies conducted in various parts of the world, including Australia [15], [26]–[28], New Zealand [29], Denmark [30], the United States [31] and Brazil [32] investigated the occurrence of *G. duodenalis* cysts in rainwater harvesting tanks. These studies found between 0% and 45% positivity for *G. duodenalis* among the samples tested. Nevertheless, few epidemiological studies have been conducted to assess health risks or protective effects associated with consumption of this water source [33]. Furthermore, there are no reports in the literature of epidemiological studies of human giardiasis associated with the consumption of harvested rainwater in developing countries.

Thus, given the scope of the P1MC Programme and considering the need to investigate its effects on the health of those who benefit, this study sought to evaluate whether access to rainwater harvesting cisterns is associated with a lower prevalence of *G. duodenalis* infections in children under five years of age, compared to children living in households supplied by other water sources.

## Methods

### Ethical considerations

This study was approved by the institutional review board of the Federal University of Minas Gerais (No. ETIC 279/09). Legal guardians of the children involved in this study were required to sign an informed consent form. Furthermore, the children were granted medical care and treatment when necessary.

### Study design and population

This study is part of a broader research project that sought to perform an epidemiological evaluation of the P1MC program to assess the impact of rainwater harvesting cisterns on different health indicators of families in rural communities. For this purpose, we chose to study children of up to five years of age, who are more susceptible to diseases facilitated by poor water supply and sanitation conditions. For the evaluation, in addition to the effect on the occurrence of *G. duodenalis* cysts in the children's feces, other outcomes used were the occurrence of other parasites and diarrhea [34]. Analyses of the microbiological quality of the water consumed by the monitored families were also performed [35].

The municipalities selected for conducting this study were Berilo and Chapada do Norte, which both are located in the Jequitinhonha Valley, in the semiarid northeast of Minas Gerais state, Brazil. These municipalities present a low human development index (HDI) with 0.680 for Berilo and 0.641 for Chapada do Norte. The index of Minas Gerais state is 0.773 and that of Brazil is 0.766 [36].

The study followed a quasi-experimental design with two concurrent cohorts, which are characterized as follows: cistern group: composed of children, aged 4 months to 5 years old, living in a rural area of the selected region, who had in their homes or were using third-party cisterns as rainwater storage systems; comparison group: composed of children, aged 4 months to 5 years old, who did not have access to a cistern. The water they used mainly originated from water sources with no sanitary protection, including rivers, springs and dams.

The sample size was established for the larger study, which included several other outcomes apart from prevalence of *G. duodenalis*. Based on an expected prevalence of the main outcome of 20% for the comparison group and 12% for the cistern group with a 95% confidence interval and 80% power of the test, an initial sample of 706 children with 353 in each group was chosen, using Epi Info software version 3.5.1. Allowing losses of 20%, this would give 565 children. Considering a prevalence of *G. duodenalis* infection of 22%, based on a range from 18 to 26% in Minas Gerais State [2], [10], this sample would be sufficient to detect a reduction of prevalence to 12.8% (RR = 1.72).

In order to form the cistern group, all the children from the two municipalities which met the established requirements for the group were selected. The children from the comparison group were selected from rural communities in the same municipalities, based on the same criteria as the cistern group, with the single exception that they did not have a cistern; in practice almost all eligible children were selected. Both groups of children were supported by the same field workers of the Family Health Program.

### Data collection

Field surveys were carried out during a period of approximately one year (2010), and the data collection relied on the support of community health workers from the Family Health Program, which is a component of the Brazilian Unified Health System. Health workers monitored families in a given geographic area, performing monthly visits to the houses of those families to prevent or monitor the population's health problems.

The first contact with the selected families occurred from September to December 2009. After being informed about the research aims and questioned on their interest to participate in the study, two families refused to participate. For the other families, after joining the study, a structured questionnaire was applied, including 55 common questions to all participants from both groups. From these, 48 produced categorical variables and 7 elicited continuous numerical variables, which referred to: (i) conditions of mother and child's health: child's sex and age, mother's age, child's hospitalization and vaccination, breastfeeding duration, vitamin supplementation, anti-parasite treatment, diarrhoea in the past 72 hours, pregnancy order, and prenatal exams; (ii) family structure: child's caretaker and caretaker's schooling; (iii) family socioeconomic conditions: number of rooms and inhabitants in the household, toilets, house characteristics (walls, roof and floor), if the family is given any financial aid from the government, and total family income; (iv) environmental and sanitary conditions of the household: place where the family defecates, destination of dirty diapers and wastewater, existence of a stream close to the household, and children's contact with the stream; (iv) solid waste and vectors: garbage destination, presence of flies, cockroaches and rats in the household; (v) child and family's hygiene conditions: frequency of children's bath, habit of washing children's hands, habit of washing the food preparer's hands; disinfection of fruit and vegetables.

To assess the impact of rainwater cisterns on children's health, monitoring forms were provided in two additional stages (middle and end) after the beginning of the data collection to verify if any family from the comparison group had received a new rainwater harvesting cistern during the year in which the children were monitored. These forms were applied by health workers in April/May 2010 and October 2010.

### Parasitological evaluation

The monitoring of intestinal parasitic diseases in the children was performed in three stages at dates close to the application of the structured questionnaires and monitoring forms. The TF-Test (Three Fecal Test) method was used. It utilizes centrifugation, filtration and sedimentation and has a high sensitivity in detecting protozoa cysts [37].This method was also selected because it allows a prolonged sample preservation time (30 days) without the need for refrigeration. In each stage, three fecal samples were collected from the same subject on alternate days in a 5 day period and placed in separate vials. This approach increased the sensitivity of the technique as appearance of Giardia in stools can be intermittent [38].

All three collection vials from each kit were identified with the child's first name to avoid confusion between fecal samples in households where more than one child participated in the research study. In the first stage, the TF-Test kits were handed out to each household during the structured questionnaires implementation. In the second and third stages, the kits were left with the health workers to be delivered to families before each specified data collection round. The health worker arranged an approximate date for sample collection with the children guardian following an orientation on the proper way of performing the sampling.

It is noteworthy that 37 children (23 in cistern group and 14 in comparison group) did not receive the TF-Test kits at Stage 1 because they were less than four months of age at the time. It was a criterion of the study not to include children up to four months of age due to the protection provided by breast milk and the very low exposure to water sources.

In Chapada do Norte, logistic field problems prevented the third stage of data collection from taking place six months after the second stage, in October 2010. The stool exams for the 416 children from the municipality, who had been monitored in stages 1 and 2, were therefore not carried out until August 2011 (16 months after the second stage). Analyses were performed by the team from the Laboratory of Parasitology, President Antônio Carlos University (Universidade Presidente Antônio Carlos; UNIPAC) in the municipality of Teófilo Ottoni, which is located approximately 270 km from Berilo and is the region's reference laboratory. The laboratory staff were blinded as to the exposure status of each child.

The results forwarded by the laboratory staff were immediately transferred to the health workers in the selected municipalities, who were responsible for providing chemotherapy to infected children.

### Statistical analysis

Generalized Estimating Equations (GEE) were used for the data analysis. Household was the first cluster specification for considering more than one child in each house. Chronological stage of the study was the second level to assess the occurrence of *G. duodenalis* in each child at each time point. A logit link function was used in order to have odds ratio interpretation for the model parameters.

Exposure variables were initially considered one by one. Out of all the categorical and continuous covariates, those showing a significance level of less than 0.20 in univariate analysis were selected for the initial model. In order to reach the final model, covariates screened in the initial model (see Supporting File – Table S1) were consecutively dropped until all of them showed a significance of p<0.05. Analyses were performed with R open-source statistical software, version 2.13.

## Results

### Characterization and comparison of the groups

The monitoring forms, filled out at stages 2 and 3, showed that the median household in the cistern group had owned their cistern for 36 months; no family from the comparison group had received a cistern at stage 2, and five families had received cisterns at stage 3. In the comparison group, 38 (11%) monitoring forms at stage 2 and 83 (25%) for stage 3 were neither returned nor filled out. It was assumed that the main water supply for these children at Stage 1 remained unaltered in all subsequent stages. In the analysis, out of 664 children monitored, 332 had rainwater cisterns and 332 did not at stages 1 and 2. However, due to these changes in exposure status these values became 337 and 327 children, respectively, at stage 3.

For characteristics evaluated, including those regarding child and mother's health, hygienic and sanitary habits and also concerning families' socioeconomic conditions, both groups are rather similar and there were no statistically significant differences when comparing them, besides the difference in the main water supply and in some factors associated with it, such as use of treated water or existence of river or stream close to the household. The groups are compared with regard to a selection of variables in Table 1.

**Table 1:** Characteristics of children from cistern group compared with children from comparison group.

Variables		Group^a^				
		Cistern group		Comparison group		p-value
		N	%	N	%	
Child's sex	Female	161	48.5	154	46.4	0.59
	Male	171	51.5	178	53.6	
Pregnancy order	First	115	34.7	116	34.9	0.96
	Second or superior	216	65.3	216	65.1	
Breastfed at least once	Yes	327	98.8	323	97.3	0.16
	No	4	1.2	9	2.7	
Prenatal monitoring during pregnancy	Yes	319	98.2	322	97.6	0.61
	No	6	1.8	8	2.4	
Duration of pregnancy	Full term	241	73.7	257	78.6	0.14
	Less than full term	86	26.3	70	21.4	
Person responsible for child's care during the last year	Mother	282	84.9	298	89.8	0.06
	Another person	50	15.1	34	10.2	
Child's caretaker's schooling	Illiterate	25	7.6	36	10.8	0.15
	Reads and writes	304	92.4	296	89.2	
Hand hygiene prior to food preparation	Always	328	98.8	324	97.6	0.24
	With small frequency or never	4	1.2	8	2.4	
House construction material	Brickwork	113	34.0	123	37.0	0.63
	Adobe (clay)	212	63.9	204	61.4	
	Wood	7	2.1	5	1.5	
Financial aid from the government	Yes	240	72.3	238	71.9	0.91
	No	92	27.7	93	28.1	
Total family income	From R$ 0.00 to 100.00	27	8.3	25	7.7	0.94
	From R$ 101.00 to 500.00	202	62.3	202	62.2	
	Above R$ 500.00	95	29.3	98	30.2	
Destination of home garbage	Burnt, buried or collected	290	87.3	281	84.6	0.32
	Open-air or thrown in the river	42	12.7	51	15.4	
Flies/mosquitoes observed in the household throughout the year	Yes	296	89.2	307	92.7	0.11
	No	36	10.8	24	7.3	

a The children's classification was considered in the first stage (332 children in cistern group and 332 children in comparison group).

Regarding the specific characteristics of the cistern group, it was found that, although 72% of the rainwater cisterns were built by P1MC, there were also cisterns built by other institutions (21%) or at family's expense (4%). For 3% of the children, the family could not confirm who was responsible for the construction of the cisterns. Most (85%) of the cisterns presented a total volume of 16,000 litres, 10% had a 25,000 litre capacity and the others presented different volumes (5%) or the participants could not answer (0.6%). It is important to point out that, at the time of the structured questionnaires application, there were cisterns built at least one month and at most ten years ago. On average, the cisterns were 33 months old.

For families in the comparison group who had not received cisterns, the reason was usually that they depended on other sources of water supply/75% reported that they used water from rivers, 23% used water from springs and 2% used water from dams or wells. For 79% of the children, the water is piped from the source to the inside of the house through an electric pump or by gravity, and for 15%, carried in a bucket or other container. For 6% of the children, the water is brought through a hose or there is a connection with the main water supply network. Most of the interviewed families (98%) claimed to use the water for several purposes, such as drinking, cooking, bathing, brushing teeth, washing dishes and clothes, and cleaning the house.

### Epidemiological evaluation of *G. duodenalis* infection

In the present study, the loss to follow-up in the parasitological examinations increased at stages 1 and 2, but only slightly at stage 3. Those losses were mostly related to the family's moving to another region (migration seeking better work conditions, which is a frequent practice among families living in Brazilian and other semiarid regions) or a failure to return the TF-Test kit with the children's feces. For the cistern group and for the comparison group these losses have been, respectively: 40 and 48 in stage 1; 93 and 105 in stage 2, and 98 and 102 in stage 3.

Comparing those children that returned at least one sample for parasitological examination during the three stages, with those who did not return any, no statistical difference was found. Table 2 shows the comparison for selected variables.

**Table 2:** Losses regarding the return of stool samples for parasitological examination.

Variables	Category	Children who returned at least one sample (n = 635)		Children who returned no sample (n = 29)		p-value
		n	%	n	%	
Cistern	Yes (Cistern group)	323	50.9	14	48.3	0.79
	No (Comparison group)	312	49.1	15	51.7	
Child's sex	Female	297	46.8	18	62.1	0.11
	Male	338	53.2	11	37.9	
Duration of pregnancy	Full term	475	76.0	13	72.2	0.70
	Less than full term	150	24.0	5	27.8	
Preparation of fruit & vegetables prior to consumption	Washed with treated water	117	18.4	6	20.7	0.12
	Washed with untreated water	402	63.3	22	75.9	
	Washed/disinfected with bleach or vinegar	116	18.3	1	3.4	
Total family income	From R$ 0.00 to 100.00	48	7.7	4	14.3	0.40
	From R$ 101.00 to 500.00	389	62.6	15	53.6	
	Above R$ 500.00	184	29.6	9	32.1	

As shown in Table 3, the prevalence of *G. duodenalis* at Stages 1 and 2 was higher for children from households without access to cisterns, but there was a reversal at Stage 3. The prevalence in children from the cistern group ranged from 4.8% to 10.5%, while the prevalence in the comparison group ranged from 7.6% to 16.7%.

**Table 3:** Prevalence of *G. duodenalis* infection in children by type of water supply and stage, Northeast of Minas Gerais State, Brazil, 2009–2010.

	Stage 1				Stage 2				Stage 3			
	Cistern group (N^a^ = 332)		Comparison group (N = 332)		Cistern group (N = 332)		Comparison group (N = 332)		Cistern group (N = 337)		Comparison group (N = 327)	
	n^b^	%	n	%	n	%	n	%	n	%	n	%
Positive	14	4.8%	31	10.9%	22	9.2%	38	16.7%	25	10.5%	17	7.6%
Negative	278	95.2%	253	89.1%	217	90.8%	189	83.3%	214	89.5%	208	92.4%
Total of exams	**292**	**100%**	**284**	**100%**	**239**	**100%**	**227**	**100%**	**239**	**100%**	**225**	**100%**

a N =  initial number of monitored children.

b n =  number of exams after losses to follow-up.

The categorical and continuous variables selected in the univariate model are presented in Tables 4 and 5 respectively. The variables that remained significant in the multivariate model after adjusting for other variables are shown in Table 6. It was observed that the odds of *G. duodenalis* infection were 1.72 times higher (95% CI 1.14-2.59) in children who did not have access to cisterns than among those who had access; therefore, cisterns were found to have a protective effect. This odds ratio is the same as the value which emerged from the power calculation, but this is entirely coincidental. The power calculation was based on a far higher assumed prevalence than was found for giardiasis. However, other variables were noticeably also independently associated with *G. duodenalis* infections. An increase in the number of rooms per house significantly reduced the odds of infection (OR 0.89; 95% CI 0.80–0.99). Regarding “birth order”, firstborn children had lower odds of infection than those from subsequent births (OR 1.72; 95% CI 1.17–2.51). The odds of infection were 1.70 (95% CI 1.19–2.43) times greater for preterm children than for others. Improper hand hygiene prior to food preparation increased the odds of *G. duodenalis* infections by 4.78 (95% CI 1.95–11.76) times. An increased family income reduced the odds of children presenting with *G. duodenalis* infections (OR 0.48; 95% CI 0.26–0.88).

**Table 4:** *G. duodenalis* infection in children in Northeast region of Minas Gerais State, Brazil, 2009–2010; **categorical** variables selected in the univariate analysis^a^.

		OR^b^	95% CI	p-value
	**Water supply**			
Type of water supply	With a cistern	ref		
	Without a cistern	1.54	1.06–2.22	0.022
	**Mother**'**s health**			
Pregnancy order	First	ref		
	Second or superior	1.46	1.01–.11	0.044
Duration of pregnancy	9 full months	ref		
	Less than 9 months	1.42	1.01–2.01	0.048
	**Family structure**			
Person responsible for child's care during the last year	Mother	ref		
	Another person	0.48	0.24–0.99	0.046
Child's caretaker's schooling	Illiterate	ref		
	Reads and writes	1.79	0.87–3.68	0.114
	**Hygiene habits**			
Hand hygiene prior to food preparation	Always	ref		
	With small frequency or never	3.50	1.63–7.53	0.001
Food hygiene prior to consumption	Washed with treated water	ref		
	Washed with untreated water	0.73	0.46–1.15	0.177
	Washed/disinfected with bleach or vinegar	1.01	0.60–1.69	0.977
	**Socioeconomic characteristics**			
Total family income	From R$ 0.00 to 100.00	ref		
	From R$ 101.00 to 500.00	0.59	0.33–1.04	0.068
	Above R$ 500.00	0.59	0.32–1.08	0.088
	**Sanitation conditions**			
River or stream close to the household	No	ref		
	Yes and the children have contact with the water	1.55	1.03–2.33	0.038
	Yes, but the children don't have contact with the water	1.04	0.7–1.55	0.834
	**Solid waste and vectors**			
Destination of household garbage	Burnt, buried or collected	ref		
	Open-air or thrown in the river	1.39	0.87–2.21	0.166
Flies/mosquitoes observed in the household throughout the year	Yes	ref		
	No	0.61	0.3–1.25	0.175

a the univariate analysis associated each independent variable with the outcome and was adjusted only by the stage (time of data collection).

b OR =  Odds Ratio.

**Table 5:** *G. duodenalis* infection in children in Northeast region of Minas Gerais State, Brazil, 2009–2010; continuous variables selected in the univariate analysis^a^.

	OR^b^	95% CI	p-valor
Number of inhabitants per household	1.05	0.98–1.13	0.148
Number of rooms per household	0.90	0.81–1.01	0.068

a the univariate analysis associated each independent variable with the outcome and was adjusted by the stage (time of data collection).

b OR =  Odds Ratio.

**Table 6:** Multivariate model for the analysis of *G. duodenalis* infection in children living in Northeast region of Minas Gerais State, Brazil, 2009–2010, according to socioeconomic, sanitary and pregnancy aspects.

	OR^a^	95% CI	p-value
Type of supply			
With cistern	ref	-	-
Without cistern	1.72	1.14–2.59	0.010
Stage			
Stage 1	ref	-	-
Stage 2	1.76	1.13–2.73	0.012
Stage 3	1.21	0.74–1.96	0.448
Birth Order			
First	ref	-	-
Subsequent	1.72	1.17–2.51	0.005
Duration of pregnancy			
9 full months	ref	-	-
Less than 9 months	1.70	1.19–2.43	0.004
Person who cooks washes his or her hands before starting activities			
Always	ref	-	-
With small frequency or never	4.78	1.95–11.76	0.001
Preparation of fruit & vegetables prior to consumption			
Washed with treated water	ref	-	-
Washed with untreated water	0.54	0.34–0.88	0.013
Washed/disinfected with bleach or vinegar	0.82	0.48–1.39	0.464
Total family income			
From R$ 0.00 to 100.00	ref	-	-
From R$ 101.00 to 500.00	0.48	0.26–0.88	0.018
Above R$ 500.00	0.61	0.32–1.13	0.117
No. rooms per house	0.89	0.80–0.99	0.034

a OR =  Odds Ratio.

## Discussion

The lowest prevalence of *G. duodenalis* infections in the present study, which was equivalent to 4.8%, was found in children from the cistern group at stage 1, while the highest value, corresponding to 16.7%, was found at stage 2 in children from the comparison group, that is, those children whose water supply originated from water sources without sanitary protection, including rivers, springs and dams. In recent studies conducted in other Brazilian regions with people from different age groups [1], [7]–[10], the prevalence of *G. duodenalis* infections ranged from 10.6% to 32.1%.

The values reported in some of the studies cited above [1], [8], [10] may be underestimated because only one fecal sample was collected per child. It is known that the release of *G. duodenalis* cysts by the host occurs intermittently; therefore, at least three samples should be collected on alternate days, as was performed for the children analyzed in this study, to increase the detection sensitivity [38], [39]. Nevertheless, Flanagan [38] warns about the possibility of a false-negative diagnosis.

In her review article, Flanagan [39] also notes that the two main risk factors associated with *G. duodenalis* infections are the consumption of unfiltered water that originated from surface water sources and, for children, placement in daycare centers. Additionally, Teixeira et al. [2] reported that the consumption of water from springs and wells significantly increased the odds of *G. duodenalis* infections in children who were one to five years old and living in informal settlement areas in Brazil.

As reported in the literature, the correlation between water consumption and pathogenic protozoa, especially *G. duodenalis*, infections has been demonstrated [2], [40]. However, the question of what sources of water supply increase the risks of infection by these protozoa is still controversial because they have been detected in surface and underground sources and in rainwater. A multivariate analysis of the present study clarifies which factors affect the odds ratio of an infection by flagellate protozoans and in which direction.

After adjusting for socioeconomic, behavioral and hygiene habit variables, the odds ratio of a *G. duodenalis* infection were found to be 1.72 times higher in the children with no access to cisterns than among those from households with cisterns. This result is supported by the sedimentation effect of *G. duodenalis* cysts that may occur within cisterns, since water remains stored over a long period. Furthermore, a regular cleaning of the tanks, if performed as recommended, may further decrease the odds of ingesting cysts. As reported in the literature, many researchers have investigated the presence of cysts from this protozoan in rainwater harvesting cisterns but have not detected them [15], [29], [30].

In this study, we have used an intention-to-treat analysis. In the event, 16 families with cisterns (5%) at Stage 2 and 38 (11%) cistern families at Stage 3 were found during the field visits to have no water in their cisterns. It is possible that the association might have been stronger if we had taken account of cistern functionality.

The results of the present study have shown a possible protective effect of rainwater harvesting systems when compared to more insecure sources of water supply. However, other studies have shown an increase in the risk of *G. duodenalis* infection by rainwater ingestion, when comparing water from cisterns with sources likely to be safer. Hoque et al. [33] found a significant odds ratio of 8.3 for people that consumed roof harvested rainwater compared to those consuming water from the main water supply network of Auckland. Omar et al. [40] also found a significant odds ratio of 2.97 for giardiasis in people that consumed rainwater, rather than desalinated water.

Other variables were also independently correlated with *G. duodenalis* infections. An increased number of rooms per house was significantly associated with reduced odds of infection, which may be related to less interpersonal contact, thereby hindering the person-to-person transmission route, as already reported by many researchers regarding *G. duodenalis* infections [33], [41], [42].

Conversely, the number of rooms per house can also be linked to the economic situation of the family; thus, the result remains consistent when considering that wealthier families have better sanitation and hygiene conditions, which decreases the odds of infection with *G. duodenalis* cysts. This hypothesis was also confirmed by a household income analysis, which found lower odds of infection in children from families earning over R$ 100.00 (about US$ 40.00) a month. Similar results were reported by Machado et al. [43], who found (in a bivariate analysis) a correlation between a low socioeconomic status, which was represented by the family income and parental educational level, and *G. duodenalis* infections.

Regarding the “birth order”, the firstborn children were confirmed to have lower odds of infection than those from subsequent births. This result may be correlated, once again, with the transmission via interpersonal contact but also with the greater care provided by mothers to their first child, minimizing the risk of infection for those children.

As expected, some hygiene habits were independently correlated with the outcome under analysis, such as improper hand hygiene prior to food preparation, which increased the odds of *G. duodenalis* infections by 4.78 times (Table 6). Proper hygienic habits are protective factors because they interrupt the fecal-oral transmission route of Giardia, as shown by Ratanapo et al. [44]. Regarding the variable “disinfection of fruit and vegetables,” lower odds of infection were unexpectedly observed for individuals using untreated water, but this outcome may have been affected by the fact that 64% of the interviewees reported using untreated water. Only 18.6% and 17.6% of interviewees said they used treated water and disinfected fruit and vegetables with bleach, respectively.

Finally, the “duration of pregnancy” was also selected in the multivariate model, which indicated a higher risk for preterm children and an association with socio-economic status. This result can be explained by the association of both giardiasis and preterm birth with low socio-economic status in Brazil [45], [46] as well as an aggravated risk of infections in general for preterm infants.

Despite the higher odds of *G. duodenalis* infections for children with no access to water stored in cisterns, analyses of the microbiological quality of the water showed that the water consumed by the participants of both groups had similar and compromised quality, given the high rates of detection of total coliforms and *Escherichia* coli [32]. Regardless of its quality, the volume of 16,000 liters of water, which was available in cisterns in close proximity to the house, presumably enables the maintenance of more appropriate hygienic habits than the habits of individuals who must travel longer routes to collect water.

Graeff et al. [47] found that the amount of available water can affect the frequency of hand washing. They state that a mother needs approximately 20 liters of water to wash her hands after using the toilet and changing diapers prior to food preparation to feed herself and the child. Thus, the volume of available water may have a greater impact on health than the quality of available water.

A major limitation of our study is that the allocation of cisterns was not a random process. As stated above, a set of selection criteria were applied, with a view to obtaining the maximum impact from the investment. These included (in descending order of importance) the lack of reliable alternative water sources, low household income, female household head, and relatively large numbers of children, disabled and elderly household members [13]. However, they were compared with a comparison group selected using the same criteria (except for having access to a cistern) and shown (Table 1) to have almost identical characteristics. Indeed, the selection of the comparison group practically exhausted the universe of eligible children in the two municipalities concerned. An additional control against bias arising from the allocation of cisterns is provided by the multivariate regression modelling (Table 6), which the only significant risk factors are among those which one would expect as biologically plausible, such as deficient hand or food hygiene.

The limitations of this study also include the difficulty in linking disease occurrence with the internal conditions of cement cisterns, which may promote or hinder the survival of *G. duodenalis* cysts. The parasite's unique physiological characteristics provide it with higher or lower odds of survival in certain locations. An analysis of the occurrence of the cysts in the water consumed by participants from both groups could also assist in the discussion of the findings. However, it was not possible to carry out such analysis because the tests for detection of *G. duodenalis* cysts in water samples are very expensive and require large volumes of water and specific equipment and reagents, absent in the municipalities where the study was conducted.

A third limitation is that the comparison group of children was not randomly selected. However, there is not much room for bias because in the event almost all eligible children were selected, because fewer than expected eligible children were available.

Despite the limitations, the results confirm that having a cistern to store rainwater is associated with a reduction of *G. duodenalis* infections in children 4 months to 5 years of age. However, other risk factors, both environmental and family-related, were independently associated with the risk of Giardia infection It follows that interventions are needed which address not only the poor water quality and access of low income rural households, but also their hygiene behaviour, particularly hand hygiene [48].

## Supporting Information

Table S1 Initial model for the analysis of *G. duodenalis* infection in children living in Northeast region of Minas Gerais State, Brazil, 2009-2010, according to socioeconomic, sanitary and pregnancy aspects and using marginal logistic regression (GEE).
(DOCX)

Checklist S1 STROBE checklist.
(DOC)
