# Supplementary material for: Reducing Occurrence of Giardia duodenalis in Children Living in Semiarid Regions: Impact of a Large Scale Rainwater Harvesting Initiative
Source: PLoS Negl Trop Dis. 2014 Jun 19;8(6):e2943. doi: 10.1371/journal.pntd.0002943 (PMC4063750; doi:10.1371/journal.pntd.0002943)
Supplement: Table S1 — Initial model for the analysis of G. duodenalis infection in children living in Northeast region of Minas Gerais State, Brazil, 2009-2010, according to socioeconomic, sanitary and pregnancy aspects and using marginal logistic regression (GEE). (DOCX) [file pntd.0002943.s001.docx]

**Table S1. Initial model for the analysis of *G. duodenalis* infection in children living in Northeast region of Minas Gerais State, Brazil, 2009-2010, according to socioeconomic, sanitary and pregnancy aspects and using marginal logistic regression (GEE). The variables shown are those of the 55 initially examined in univariate regression with a significance value of p<0.20.**

|  | **Β** | **SE(β)** | **wald** | **O.R** | **95% CI** | **Pvalue** |
| --- | --- | --- | --- | --- | --- | --- |
| Type of supply |  |  |  |  |  |  |
| With cistern |  |  |  | ref |  |  |
| Without cistern | 0.474 | 0.241 | 3.861 | 1.61 | 1 – 2.58 | 0.049 |
| Stage |  |  |  |  |  |  |
| Stage 1 |  |  |  | ref |  |  |
| Stage 2 | 0.554 | 0.225 | 6.072 | 1.74 | 1.12 – 2.7 | 0.014 |
| Stage 3 | 0.145 | 0.250 | 0.336 | 1.16 | 0.71 – 1.89 | 0.562 |
| Birth order |  |  |  |  |  |  |
| First |  |  |  | ref |  |  |
| Subsequent | 0.511 | 0.211 | 5.839 | 1.67 | 1.1 – 2.52 | 0.016 |
| Duration of pregnancy |  |  |  |  |  |  |
| 9 full months |  |  |  | ref |  |  |
| Less than 9 months | 0.488 | 0.188 | 6.740 | 1.63 | 1.13 – 2.35 | 0.009 |
| Person responsible for child’s care during the last year |  |  |  |  |  |  |
| Mother |  |  |  | ref |  |  |
| Another person | 0.463 | 0.359 | 1.663 | 0.63 | 0.31 – 1.27 | 0.197 |
| Child’s caretaker’s schooling |  |  |  |  |  |  |
| Illiterate |  |  |  | ref |  |  |
| Reads and writes | 0.685 | 0.402 | 2.913 | 1.98 | 0.9 – 4.36 | 0.088 |
| Hand hygiene prior to food preparation |  |  |  |  |  |  |
| Always |  |  |  | ref |  |  |
| With small frequency or never | 1.628 | 0.513 | 10.049 | 5.09 | 1.86 – 13.93 | 0.002 |
| Food hygiene prior to consumption |  |  |  |  |  |  |
| Washed with treated water |  |  |  | ref |  |  |
| Washed with untreated water | 0.581 | 0.245 | 5.614 | 0.56 | 0.35 – 0.9 | 0.018 |
| Washed/disinfected with bleach or vinegar | 0.123 | 0.278 | 0.196 | 0.88 | 0.51 – 1.52 | 0.658 |
| Floor of the house |  |  |  |  |  |  |
| Ceramic or cemented |  |  |  | ref |  |  |
| Clay | 0.485 | 0.293 | 2.744 | 0.62 | 0.35 – 1.09 | 0.098 |
| Total family income |  |  |  |  |  |  |
| From R$ 0.00 to 100.00 |  |  |  | ref |  |  |
| From R$ 101.00 to 500.00 | 0.761 | 0.326 | 5.461 | 0.47 | 0.25 – 0.88 | 0.019 |
| Above R$ 500.00 | 0.615 | 0.367 | 2.812 | 0.54 | 0.26 – 1.11 | 0.094 |
| River or stream close to the household |  |  |  |  |  |  |
| No |  |  |  | ref |  |  |
| Yes and the children have contact with the water | 0.226 | 0.246 | 0.844 | 1.25 | 0.77 – 2.03 | 0.358 |
| Yes. but the children don’t have contact with the water | 0.144 | 0.231 | 0.390 | 1.16 | 0.73 – 1.82 | 0.532 |
| Destination of household garbage |  |  |  |  |  |  |
| Burnt. buried or collected |  |  |  | ref |  |  |
| Openair or thrown in the river | 0.249 | 0.271 | 0.847 | 1.28 | 0.75 – 2.18 | 0.357 |
| Flies/mosquitoes observed in the household throughout the year |  |  |  |  |  |  |
| Yes |  |  |  | ref |  |  |
| No | 0.337 | 0.368 | 0.838 | 0.71 | 0.35 – 1.47 | 0.360 |
| Number of inhabitants per household | 0.063 | 0.043 | 2.120 | 1.07 | 0.98 – 1.16 | 0.145 |
| Number of rooms per household | 0.147 | 0.060 | 5.921 | 0.86 | 0.77 – 0.97 | 0.015 |
